# Life Skills Needs Assessment among Iranian Immigrant Students in Malaysia

**Published:** 2017-01

**Authors:** Marjan MOHAMMADZADEH, Hamidin AWANG, Esra TAJIK

**Affiliations:** 1. Dept. of Community Health, Faculty of Medicine and Health Sciences, Universiti Putra Malaysia, Selangor, Malaysia; 2. Dept. of Psychiatry, Faculty of Medicine and Health Sciences, Universiti Putra Malaysia, Selangor, Malaysia; 3. Dept. of Nutrition and Dietetics, Faculty of Medicine and Health Sciences, Universiti Putra Malaysia, Selangor, Malaysia

## Dear Editor-in-Chief

Moving on from a city to another is a stressful situation. Emigration to another country on the other hand, could be even more stressful for many reasons such as the new cultural and educational environment (1), especially for children and adolescents due to leaving a familiar environment to unfamiliar one which can seriously affect their both mental and physical health status (2). Therefore, one of the challenges for the immigrate parents to help their children to stay on correct pathways for a successful and healthy life is finding educational methods relying more on influence than control to help them adjusting their new situation (3).

life skills education (LSE) is a comprehensive method focusing on the development of the skills that people normally need for healthy life such as making decisions, controlling emotions, communication, life and relationship management, self– esteem building and more (4). Indeed, just like any other program, one of the most vital components in developing a successful training plan including LSE program, to respond the special situation of Iranian emigrant children and even their families is the education need analysis (5).

This phenomenology qualitative study aimed to reach a common understanding of life skills’ needs for Iranian immigrant students in Malaysia from the parents’ viewpoint. Fifteen mothers, aged 36–48 with different educations levels, having children aged 13–18 years old and willing to participant in the study were divided to 3 small discussion groups to discuss about study’s objectives. Four of them were secondary school teachers with more than 3 years of experience working in Iranian schools in Malaysia. Data collection was done thorough field notes and focus discussion sessions ranged between 45 and 90 min, were conducted under first authors’ supervision. Group discussions were continued until reaching Data Saturation.

During discussion sessions, participants were asked to respond to the following questions: 1- Generally, what is the description of life skills for adolescents from your viewpoint? 2- What are the life skills needed for Iranian adolescents in Malaysia referring to all differences between Iran and Malaysia? 3- What are the three most important life skills that we should teach them?

As a result, this study developed a unique parent-centered definition of a variety of daily life skills needed for Iranian adolescents to help them thrive in their future challenges. Participants in the study reached to a single definition of life skills: ‘Life skills are a variety of comprehensive skills helping us deal and cope with the daily routines and sudden life challenges. It is not important how old we are or what our position is in life, we need them to have a better and more comfortable life.

The second significant finding of this study was the list of life skills needed from mothers’ viewpoint. Doubtless, immigrant children and adolescents needs are mostly the same as their peers, but they also need compensatory skills training for adjustment to their new unfamiliar culture and environment (6).

**Fig. 1:**
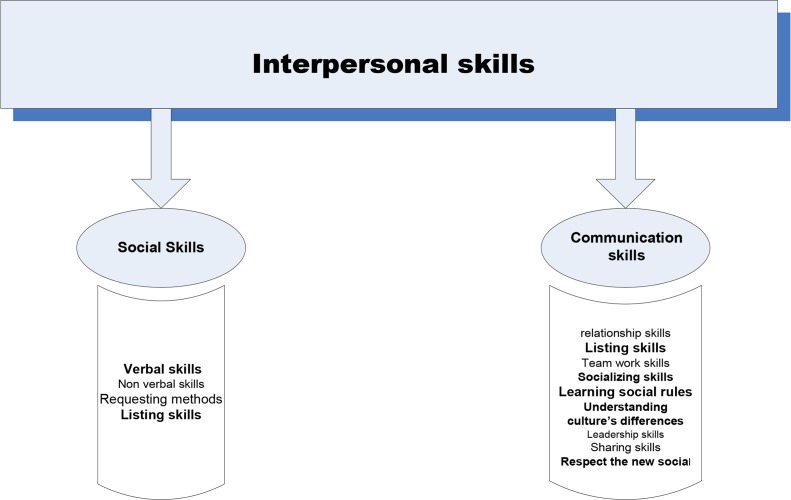
Essential interpersonal life skills for immigrant Iranian students from participants’ viewpoint

In intrapersonal skills area, community and social skills were selected as the most important skills. Verbal and non-verbal communication skills, asking methods, listening skills, relationship skills, teamwork skills, the skills to understand differences were some of the sub- skills mentioned by participants (Fig. 1).

**Fig. 2:**
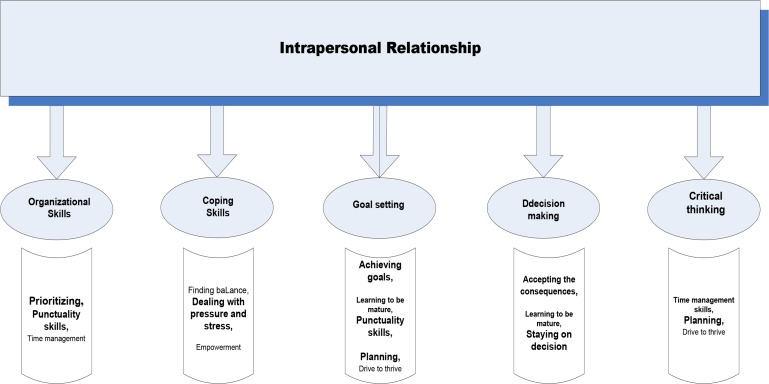
Essential intra-personal life skills for immigrant Iranian students from participants’ viewpoint

In interpersonal skills area, decision making, goal setting, critical thinking, coping with emotional and organization skills were named as the important intra personal skills for the adolescents (Fig. 2).

Current research, in its third achievement, presented the priority of life skills needed among Iranian adolescents in Malaysia. According to the results communication skills, self-organization and goal setting respectively are the three prior life skills among the immigrant adolescents.

Surly, need assessment is the first step in decision making process for any educational program including life skills training, but like other plans, it needs practice and perseverance to reach its maximum efficiency. Since, there is no special program in none of the Iranian schools in Malaysia to help students having better adjustment with their new situation; the results of the current study could be used as the primary data for planning continuous educational programs based on life skills education among Iranian students in Malaysia. These findings also could be useful for educational planners, managers and teachers to prepare immigrant adolescents for better future in a foreign country.
